# Willingness to join community-based health insurance and its associated factors among households in Nekemte City, Ethiopia. A community-based cross-sectional study

**DOI:** 10.1186/s41043-024-00553-z

**Published:** 2024-05-20

**Authors:** Olkeba Begna, Habtamu Fekadu Gemede, Aboma Motuma, Tesfaye Shibiru, Temesgen Tilahun, Firew Tekle Bobo, Meseret Belete Fite

**Affiliations:** 1 Oromia regional health bureau, Addis Abeba, Ethiopia; 2 Department of Food Technology and Nutrition, Wollega University, P.O. Box 395, Nekemte, Ethiopia; 3 School of Nursing and Midwifery, College of Health and Medical Sciences, Haramaya University, Harar, Ethiopia; 4 School of medicine, Institute of Health Sciences, Wollega University, Nekemte, Ethiopia; 5 Department of Public Health, Institute of Health Sciences, Wollega University, Nekemte, Ethiopia

**Keywords:** Household, Community-based health insurance, Nekemte city, Ethiopia

## Abstract

**Introduction:**

Ethiopia has been implementing community-based health insurance programs since 2011 to improve health care financing system. However, the prevalence of household willingness to join the community-based health insurance (CBHI) program and its associated factors are less explored in urban area. Therefore, this study was aimed to assess the prevalence of willingness to join community-based health insurance program and its associated factors among households in Nekemte City, Ethiopia.

**Methods:**

A community-based cross-sectional study was conducted on 422 randomly selected households in Nekemte City, Ethiopia. Bivariate and multivariable analyses were performed to see the association between the independent and outcome variables using binary logistic regression model. Association was described using an adjusted odd ratio (AOR) and a 95% confidence interval (CI). Finally, p-value < 0.05 was considered the cut-off point for declaring a significant.

**Results:**

Among 422 study participants, 320 (75.83%) [95% CI = 71.5-79.8%)] of the households were willing to join community-based health insurance program. The willingness to join for community-based health insurance was 3.11 times more likely among households who were in the richest quintile (AOR = 3.11; 95% CI = 1.08–8.93), 3.4 times more likely among those who were merchants (AOR = 3.40;1.33, 8.69), 2.52 times more likely among those who had history of chronic illness in the household (AOR = 2.52; 95% CI = 1.43–4.45), 4.09 times more likely among those who had the awareness about the scheme (AOR = 4.09; 95% CI = 1.97–8.47) and 3.29 times more likely among those who had the experience of borrow for medical care (AOR = 3.29; 95% CI = 1.48–7.30).

**Conclusion:**

Nearly three fourth of the households were willing to join community-based health insurance program, however, about one fourth of households were not willing, which is a significant public health problem. Being merchant, having awareness about the scheme, being in the richest wealth quintile, having experience of borrowing for medical care, and having history of chronic illness in the household were factors found to be significantly associated with willingness to join community based health insurance in the study area. Therefore, strengthening awareness creation at community level about the benefit package and principle of the program would increase their demand for the community-based health insurance scheme.

## Introduction

In the poor socioeconomic group of the community, out-of-pocket health costs fallouts into substantial financial obstacles and underprivileged life in families [1]. Thus, people with low income in poor countries yet hurt and lost their life from health-related problems mainly in areas that have no effective health insurance policies, programs, and strategies [2]. Therefore, stirring away from out-of-pocket charges for health care when practice is a significant footstep towards forestalling the economic misfortune related to paying for health care service [3]. Worldwide, nearly 44 million households (over 150 million people) face economic snags owing to medical expenditures. Subsequently, almost a 25 million households are in profound poverty each year [4].

Low- and Middle-income Countries (LMICs) are dealing with the triple burden of infectious and non-infectious diseases in a poor environment, and resource constraints which is leading to a major concentration of risk for high mortality. Preventable communicable diseases continue to be a significant public health issue in low-income countries including Ethiopia [5]. Even though Ethiopia is working toward achieving sustainable millennium developmental goals (SDGs), low health-seeking behavior and access to modern health care are still the major concerns [6, 7]. However, various factors could contribute to the lowered utilization of contemporary healthcare services, the user fee charges are one of the reasons [8]. User fees may result in a significant psychological and economic problems to the households. It is one of the obstacles to medical service particularly for poor families who are themselves likely to be especially vulnerable to ill health [9]. Although Ethiopia has been implementing CBHI programs since 2011 to improve health care financing system throughout the country [10, 11] the existing evidence focuses on rural area and less explored in urban area. In addition, since of variances in community participation with the CBHI scheme and differences in administrative and healthcare service provider facilities across different areas of the country, examining the present study area is fundamental to providing tailored comprehensions because little is acknowledged about the level of willingness to enroll for health insurance by households and its associated factors in the study area [12]. This study, therefore, aimed to assess the prevalence of willingness to join CBHI and its associated factors in Nekemte City, Western Ethiopia.

## Methods

### Study settings

A community-based cross-sectional design was conducted among households in Nekemte city, western Ethiopia. Nekemte is located 317 km Western of Addis Ababa, the capital of Ethiopia. Based on the population projection, the town has a total population of 135,856. It is the capital city of the East Wollega Zone of Oromia Regional State. A town’s altitude ranges from 1960 to 2170 m above sea level where its average annual rainfall is 1854.9 mm and the average temperature ranges from 14^O^_C_ to 26^O^_C_. There are 28,302 HHs. There are totally four [4] governmental and sixty one (61) private health institutions in this town. These include 2 health centers, 2 compressive specialized hospitals, 21 private medium clinics, 16 private drug shops, and 11 private primary clinics [13]. The coverage of the population’s access to the health center is 86.4% (Nekemte city health report 2020). The study was conducted from January 5 to February 12, 2021.

### Study design

A community-based cross-sectional study was conducted.

### Population

All Households, which were registered as permanent residents of Nekemte city during the study period were the source population. The study population was all households which were registered as permanent residents of selected kebeles of Nekemte city during the study period, whereas all households which were registered as permanent residents participated in the study period were the study unit.

### Inclusion and exclusion criteria

All Households, which were registered as permanent residents of Nekemte city were included in this study. However, household heads were not able to communicate and Households with heads or spouses that had been employed in the formal sectors were excluded from the study.

### Sample size and sampling technique

The sample size was determined using single and double population proportion formulas with their corresponding assumption, and the largest sample was considered. As such, the sample computed using single population proportion formula with the following assumptions gave the largest sample (*n* = 401): 95% confidence interval, the proportion of households were willing to join CBHI scheme(78%) in the Jimma Zone, Ethiopia [14] using 95% confidence interval (Z = 1.96) and 5% margin of error. After considering a design effect of 1.5 and adding 10% for potential non-response rate, the sample size was 422. The double population proportion assumption formula was used to calculate the sample size using Epi-Info software version 7. The following parameters were considered: a precision of 5% at a 95% confidence level and a power of 80%. The ratio of controls to cases (r) = 2, OR = 2.79, P1 = 29.5%, and P2 = 17.2% [15]. In addition, a 10% possible non-response rate was multiplied by the design effect of 1.5. Finally, a sample size of 327 (109 cases and 218 controls) was obtained. Since the largest sample size was, the sample size computed with single population proportion formula the final sample size was 422.A multi-stage sampling technique was used to select study participants. At the first and second stages, the two primary health care units (PHCU) ((Nekemte PHCU and Chlalaki PHCU) and six kebeles (three from each PHCU) were randomly selected using a lottery method out of the city and also proportional allocation was utilized to determine the number of households from each district and kebele. List of households obtained from each kebele’s administration household record list which was used as a sampling frame. The sample size was proportionally allocated for selected kebeles based on each kebele’s number of households. Finally, simple random sampling was employed to select study participants by their name using computer-generated random numbers in an Excel spreadsheet (Microsoft Corporation, 2013) from the sampling frame household.

### Data collection and measurement

Data were collected through interview administered questionnaires by trained research assistants. The questionnaire contained data on socio-economic, willingness to join the CBHI scheme knowledge and attitude. Structured questionnaires that are adopted from the review of literature were initially prepared in the English language and were translated to the local language (Afan Oromo) by an individual with good command of both languages. It was also pre-tested on 10% of the sample before data collection. The primary outcome of interest was willingness to join CBHI and the secondary outcome was estimates of factors associated with willingness to join. Willingness to join: the willingness of household heads to join the proposed CBHI regardless of the amount of payment [16] .Dependent variables were the level of willingness to join CBHI among households of Nekemte town. Independent variables included demographics (age, sex, religion, marital status, family size, ethnicity), socioeconomic variables (wealth status, occupation, education status), health and health-care utilization (illness experience within the past three months, seeking treatment, place of treatment, medical expense and coping mechanism of expenditure, chronic illness, and disabilities) as well as exposure to, and perception of, the schemes (awareness about the scheme, information source, participation in indigenous insurance scheme).

Wealth index estimated the economic level of families; the principal component analysis generated the wealth index. The index was calculated based on latrine ownership, agricultural land ownership and size, selected household assets, quantity of livestock and water source used for drinking (included 41 household variables). Willingness to join the CBHI scheme knowledge was gauged using 16 CBHI knowledge questions about CBHI. The highest tertile was defined as having “good” CBHI knowledge and the two lower thirds were labeled as “poor” CBHI knowledge. The attitude was evaluated with 12 Likert scale questions using PCA. The factor scores were totaled and classified into tertiles (three parts: the highest tertile were defined as having a “favorable” attitude and the two lower tertiles were characterized as an “unfavorable” attitude.

### Data quality assurance

Two days of rigorous and extensive training was given for data collectors and supervisors on objective of the study, duration of data collections, contents of tools, how to collect the data from the respondents’, how to fill the questionnaires and ethical consideration before the pre-test. Questionnaires were pre-tested on 10% of the sampled pregnant women of the Sire town, that were not included in the main study, and modification was done based on the pre-test observations Collected data was checked by supervisors before being sent to the data entrée on daily basis. The supervisors kept the alleyway of the field procedures and checked the completed questionnaires daily to approve the accuracy of the data collected, and the research team managed the overall work of data collection.

### Data processing and analysis

Data were double entered using EpiData Version 3.1 software. Data were cleaned, coded, and checked for missing data and outliers, for further analysis exported to STATA version 14 (College Station, Texas 77,845 USA). Descriptive statistics such as mean, standard deviation, median, frequency, percentiles and percentage were used to present socio-demographic characteristics. Bivariate and multivariable analyses were performed to see the association between the independent and outcome variables using binary logistic regression model. The assumptions for binary logistic regression were checked. The Hosmer-Lemeshow statistical test for goodness of fit and the omnibus test were performed. All variables with p-values < 0.25 in the bivariate analysis were included in the multivariable analysis final model to control all possible confounders. Multico-linearity test was performed to determine the correlation between independent variables using the standard error and collinearity statistics (variance inflation factors > 10 and standard error > 2 was considered suggestive of existence of multi co-linearity). The direction and strength of statistical association was measured by odds ratio with a 95% CI. Adjusted odds ratio along with a 95% CI was estimated to identify factors associated with folate deficiency. Pearson Correlation Coefficient checked the correlation between independent variables. A p-value < 0.25 was used as a cut-off point to select variables for the final model. Backward elimination was used with p-value < 0.05 considered statistically significant.

### Ethical consideration

All methods of this study were conducted in accordance with the Declaration of Helsinki-the Ethical principles for medical research involving human subjects. A letter of ethical approval was obtained from Addis Ababa Medical and Business College Institutional Research Ethic and Review Committee prior to beginning data collection. Written informed consent to participate was obtained from participants and their privacy and confidentiality were maintained. All personal identifiers were excluded, and data was kept confidential and used only for the proposed study.

## Results

### Socio-demographic characteristics of the respondents

A total of 422 households consented, making a response rate of 100%. The mean age of the study participants was 24.97 (+ 5.0), ranging from 18 to 78. The majority of respondents were high school and above (70.38%), were married (93.20%), and were of family size from 1 to 5 (72.27%) (Table 1).

**Table 1:** Socio-demographic characteristics of the respondents in Nekemte City, Oromia Regional State, Western Ethiopia, (*n* = 422)

Variables		Frequency (%)
Age category of the respondents		
18–24 25–32 33–39 ≥ 40		24(5.69) 105(24.88) 190(45.02) 103(24.41)
Mean of age ( *±* SD)		24.97 ± 5.00
Sex of the respondents		
Male		346(82.00)
Female		76(18.00)
Educational level of the respondents		
Cannot read and write		25(5.92)
Elementary School		10(23.70)
High school and above		287(70.38)
Marital status of the respondents		
Single		9(2.10)
Married		393(93.20)
Divorced		31(7.35)
Widowed		11(2.50)
Family size		
1–5		305(72.27)
> 5		117(27.73)
Occupational status of the respondents		
Daily labor		251(59.48)
Housewife		25(5.93)
Merchants		146(34.59)
Wealth Index (Quintile)		
Poorest Poor Middle Rich Richest		90(21.33) 81(19.19) 79(18.72) 85(20.14) 87( 20.62)

### Willingness to join CBHI

**Fig. 1:**
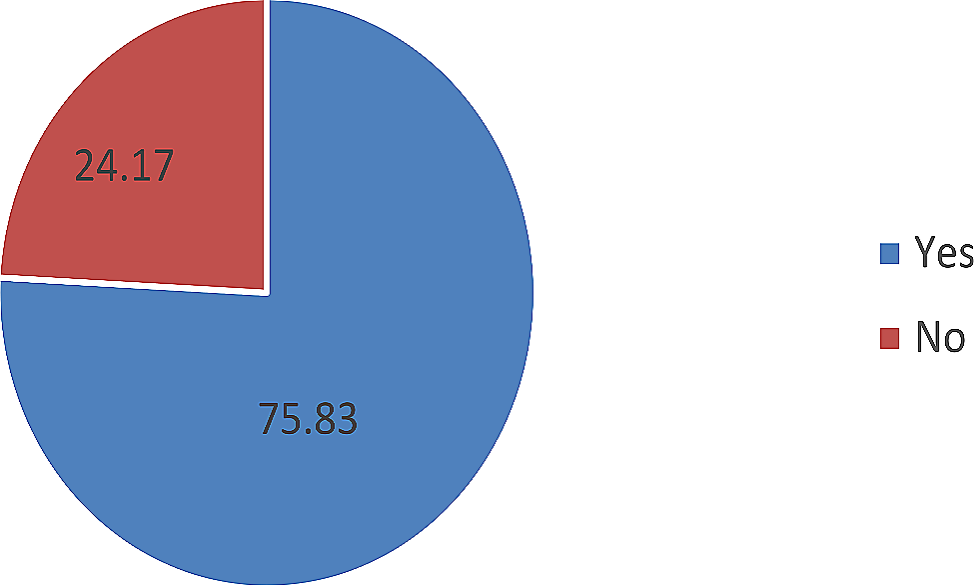
Willingness to join community-based health insurance of respondents in Nekemte Town, Western Ethiopia (*n* = 422)

Among 422 study participants, 320 (75.83%) [95% CI 71.5%-79.8%)] of the households were willing to enroll community-based health insurance program (Fig. 1).

### Factors associated with willingness to join CBHI

In the bi-variable analysis, wealth, trust scheme management, occupational status, history of chronic illnesses in the household, seeking and getting medical care, experience of borrowing for medical care, awareness about the scheme, and educational level of the respondents were found to be candidates for multivariable analysis at a p-value < 0.25. In the multivariable logistic regression model, Awareness about the scheme, wealth, experience of borrowing for medical care, occupational status, and history of household chronic illness showed a p-value < 0.05 and a significant association with willingness to join community-based health insurance.

Respondents with the richest quintile of wealth index were 3.11 times (AOR = 3.11;95% CI = 1.08–8.93), who were merchants were 3.40 times (AOR = 3.40; 95% CI = 1.33–8.69), had a history of chronic illness in the household were 2.52 times (AOR = 2.52;95% CI = 1.43–4.45), who had the e awareness about the scheme were 4.09 times (AOR = 4.09;95% CI = 1.97–8.47) and had experience of borrowing for medical care were 3.29 times (AOR = 3.29; 95% CI = 1.48–7.30) more likely to have the willingness to join CBHI, Table 2.

**Table 2:** Factors associated with willingness to join community based health insurance in Nekemte Town, Western Ethiopia (*n* = 422)

Variables	Willingness to join CBHI		COR (95%CI)	AOR (95%CI)	*P*-value
	Yes *n*(%) = 320(75.83)	No *n*(%) = 102(24.1)			
Wealth index (Quintile)					
Poorest	72(22.50 )	18(17.65)	1	1	
Poor	61(19.06)	20(19.61)	0.76(0.37,1.57)	1.62(0.68, 3.84)	0.035
Middle	54(16.88)	25(24.51)	0.54(0.27,1.09)	1.07(0.43,2.66)	
Rich	54(16.88)	31(30.39)	0.43(0.22,0.86)	0.71(0.31,1.66)	
Richest	79(24.69)	8(7.84)	2.47(1.01,6.02)	3.11(1.08, 8.93)	
**Trust scheme management**					
No	126(39.38)	41(40.20)	1	1	0.082
Yes	194(60.62)	61(59.80)	1.03(0.66,1.63)	1.74(0.93,3.26)	
**Being merchant**					
No	*181(56.56)*	95(93.14)	1	1	
Yes	139(43.44)	7(6.86)	10.00(4.69,23.17)	3.40(1.33, 8.69)	0.010
**History of chronic illness in the household**					
No	85(26.56)	61(59.80)	1	1	
Yes	235(73.44)	41(40.20)	4.11(2.58,6.56)	2.52(1.43,4.45)	0.001
**Seeking and get medical care**					
No	112(38.75)	35(34.65)	1	1	
**Yes**	177(61.25)	66(65.35)	0.84(0.52,1.35)	1.070(0.61,1.89)	0.805
**Experience of borrow for medical care**					
No	221(69.06)	92(90.20)	1	1	
**Yes**	99(0.94)	10(9.80)	4.12(2.06, 8.25)	3.29(1.48,7.30)	0.003
**Awareness about the scheme**					
No	137(42.81)	87(85.29)	1	1	
**Yes**	183(57.19)	15(14.71)	7.75(4.29,13.99)	4.09(1.97,8.47)	0.000
**Educational status of the respondents**					
Cannot read and write	19(5.94)	6(5.88)	1	1	
Elementary School	91(28.44)	9(8.82)	3.19(1.02,10.04)	1.53(0.37,6.31)	0.559
High school and above	210(65.63)	87(85.29)	0.76(0.29, 1.97)	1.03(0.32,3.33)	

## Discussion

This study investigated the status of willingness to join community based health insurance and its associated factors in Nekemte Town. The study revealed that 75.83% respondents were willing to join community-based health insurance. Among several variables, awareness about the scheme, wealth, experience of borrowing for medical care, occupational status, and history of chronic illnesses in the household were significantly associated with willingness to join community-based health insurance.

The prevalence of household’ willingness to join community-based health insurance observed in this study was comparatively lower than in the previous national study that reported 80% [16] and other studies conducted in East Gojjam (81.5% [17]. However, the current finding is consistent with the carried out in the rural households of selected districts of Jimma Zone (78%) [18] and in the Gemmachisdistrict, eastern Ethiopia (74.8%) [19]. However, due to the differences in the study area and socio-cultural conditions, it is noteworthy that the direct comparison of our results with previous studies in Ethiopia is impossible.

In this study, household wealth status was positive and significantly associated with willingness to join community-based health insurance. This result is consistent with previous studies reported in different parts of the countries [20–23] and Ethiopia [24]. However, this result is comparatively inconsistent with another study conducted Boricha District, Sidama Zone, Southern Ethiopia [25] which reported, the household wealth index is not significantly associated with direct enrollment in CBHI. This could be due to the socio-economic and cultural background differences of the study populations. Wealth is related to high asset losses if an unexpected event occurs that leads to households more willing to join community-based health insurance than the poorer one. The possible explanation for this inconsistency may be due to the study area where Boricha is the rural district and almost all households in the study area had cultivated lands and owned common domestic animals [25]. This suggests that there may not be as such an economic difference between households. Therefore, these reasons may have influenced the association of the household wealth index with CBHI registration.

Additionally, factors related to the health facilities, such as the access to information about CBHI, also play crucial roles in determining CBHI membership [26]. The current study revealed that being informed about the scheme was significantly associated with the willingness to join for community-based health insurance. This is consistent with reports from studies conducted in Nigeria [27] and Ethiopia [25]. This could be due to understanding the terrible effects of health problems and the benefit of joining the insurance scheme earlier. It is possible; people with adequate information can request details of the services and gain better understanding of its benefit which motivates them to join community-based health insurance. It is found that household members’ medical history of illness in the last three months had significant effect on willingness to join community-based health insurance. This result is consistent with other studies in India [21], Bangladesh [16], and Ethiopia [28, 29]. This could be because risk-averse individuals are more likely to willing to join community-based health insurance. Similarly this result was supported by study conducted in South Gondar Zone, Amhara, Ethiopia [2] reported that Households without chronic illness family members were 0.37 times less likely to be CBHI health care service utilizers as compared with households without chronic illness family members while holding other covariates constant. This show chronic illness membered households were more likely CBHI healthcare service users. This may be due to households with chronic illnesses members being more likely to be admitted to the hospital. Because more hospitalization has financial consequences, they are more likely to seek CBHI healthcare **services.** This study contradicts the previous study [20] shows households that have recorded sick members are less willing to pay to enroll in CBHI, and [30] shows that the presence of chronic illness family members had no significant effect on household’s enrolment in CBHI. The difference might be due to sociodemographic, the time and study setting, the scheme implementation rule, and quality of health care difference.

In addition, the occupational status of the respondents also affects their willingness to join for community-based health insurance. Merchants are more likely had willing to pay more for health care. The possible reason for this might be merchants have better income to earn fees when they get sick.

## Conclusion

About one fourth of households in Nekemte city had not willing to join community-based health insurance. Being merchant, having awareness about the scheme, being in the richest wealth quintile, having experience of borrowing for medical care, and having history of chronic illness in the household were positively significant associated with willingness to join community based health insurance. Therefore, in order to make the CBHI scheme more attractive to all citizens with different socioeconomic status, at least in the short term, the premium for membership should be customized by individual socioeconomic factors. In addition, strengthening awareness creation at community level about the benefit package and principle of the program would increase their demand for the CBHI scheme.

## Data Availability

All data are available within the manuscript. Additional data can be obtained from the corresponding author with a reasonable request.

## Abbreviations

CBHI: Community Based Health Insurance
